# A nationwide survey on the implementation of infection prevention and control components in 1442 hospitals in the Republic of Korea: comparison to the WHO Infection Prevention and Control Assessment Framework (IPCAF)

**DOI:** 10.1186/s13756-022-01107-w

**Published:** 2022-05-13

**Authors:** Yoolwon Jeong, Hyeyoung Joo, Hyunjung Bahk, Hyunsuk Koo, Hyungmin Lee, Kinam Kim

**Affiliations:** 1 Ministry of Health and Welfare, Sejong, 30113 Republic of Korea; 2 Korea Disease Control and Prevention Agency, Cheongju, 28159 Republic of Korea

**Keywords:** Infection control, Survey, COVID-19, Republic of Korea

## Abstract

**Background:**

The current SARS-CoV-2 pandemic continues to underscore the inadequacy of infection prevention and control (IPC) and the importance of its sound establishment in healthcare facilities. The Infection Prevention and Control Assessment Framework (IPCAF) by the World Health Organization allows systematic assessment of IPC capacity in healthcare facilities and has been applied in many national-level surveys. This study aims to assess the IPC capacity of Korean hospitals as well as their strengths and pitfalls by analyzing the results of the first government-led nationwide IPC survey in comparison to the IPCAF frame.

**Methods:**

The Korean National Infection Prevention and Control Survey (KNIPCS) was conducted from February to March 2018. The survey questionnaire for KNIPCS was developed through a series of expert consultations and a round of pre-testing in two randomly selected hospitals. The survey questionnaire was distributed to a total of 2108 hospitals. Although the survey preceded the release of IPCAF, its contents complied with IPCAF to a large extent, allowing exploration of its results with regards to IPCAF.

**Results:**

All tertiary hospitals and 96.5% of general hospitals had implemented IPC teams, whereas the percentage was lower for long-term care hospitals (6.3%). A similar trend was observed for IPC surveillance and monitoring activities across hospital types. The percentage of interactive IPC training was lower than 30% in all hospital groups. Disinfection was frequently monitored in all hospital types (e.g. 97.3% in general hospitals and 85.3% in long-term care hospitals). However, activities regarding antimicrobial resistance, such as multi-drug resistant pathogen screening, were weak in hospitals (25%) and long-term care hospitals (25%), compared to tertiary hospitals (83.3%) and general hospitals (57.7%).

**Conclusions:**

In general, essential IPC structures, such as IPC teams and programs, were well in place in most tertiary and general hospitals in Korea. These hospital groups also actively conducted various IPC activities. As most previous legislative and multimodal policy measures have targeted these hospital groups, we speculate that future policy efforts should encompass long-term care hospitals and smaller-sized hospitals to strengthen the IPC capacity of these hospital groups. Efforts should also be put forth to promote IPC training and antimicrobial activities.

**Supplementary Information:**

The online version contains supplementary material available at 10.1186/s13756-022-01107-w.

## Background

Healthcare-associated infection (HCAI) and infection prevention and control (IPC) have emerged as one of the most significant public health issues worldwide. Meanwhile, the current SARS-CoV-2 pandemic is underscoring the inadequacy of IPC and the importance of its sound establishment in healthcare facilities. For optimal and timely administration of IPC practices in disease outbreak situations, a dedicated team of IPC specialists and a facility-level IPC program should be up and running in advance. It is evident that these essential IPC structures are critical in securing basic IPC in facilities and guaranteeing minimum protection to their patients and staff [1]. Since 2016, the World Health Organization (WHO) has recommended IPC teams and in-facility IPC programs as one of the core components of IPC [2], and again highlighted its significance in the coronavirus disease IPC guidance in 2021 [3].

In Korea, despite the advancement in medical technology and quality of healthcare services, HCAI has evolved into a serious health concern with a significant socioeconomic burden [4]. In response to such increased public concern, a series of relevant policies and strategies have been rolled out to strengthen IPC capacity of healthcare facilities. These policy measures included legislative approaches that mandated hospitals to operate IPC teams and programs, as well as various quality-driven approaches such as improvement in systems and the built environment, positive reinforcement through incentives and reimbursement, and performance evaluation programs, among others [5]. In early 2018, the government developed and executed the Korean National Infection Prevention and Control Survey (KNIPCS) as a means to gain a detailed picture of the extent to which IPC structures and capacity were strengthened in Korean hospitals as a result of these policy measures.

In September 2018, the WHO released the Infection Prevention and Control Assessment Framework (IPCAF), which is a structured tool that allows systematic assessment of IPC capacity in healthcare facilities [6]. It is based on the “Guidelines on Core Components of Infection Prevention and Control Programmes”, and thus consists of questionnaires pertaining to each core component (CC) of the guidelines. The tool is primarily intended to be used as a self-assessment by facilities, but can also be used for the purpose of joint assessment between an external assessor and the facility. Accordingly, analysis of nationwide assessments and inter-country comparisons using the IPCAF was applied in numerous previous studies, which have provided valuable insights [7, 8]. Although the KNIPCS did not directly apply the IPCAF questionnaire, its contents comply with the IPCAF to a large extent, allowing exploration of its results with regards to IPCAF. This study aims to assess the IPC structure and programs of Korean hospitals, as well as their strengths and pitfalls, by analyzing the results of the KNIPCS in comparison to the WHO IPCAF frame.

## Methods

The KNIPCS was conducted from February to March 2018 by the Ministry of Health and Welfare and the Korea Disease Control and Prevention Agency. As the questions in the survey were developed to examine the status of IPC in hospital-level institutions, the survey targeted hospitals only and excluded clinic-level institutions. According to the Korean Medical Service Act, a clinic-level institution is defined as a medical institution that primarily provides outpatient services. Clinic-level institutions currently do not have a legal obligation to operate IPC teams or committees, and therefore were excluded from the survey. A survey focal point in respective hospitals, who were either IPC nurses or hospital administrative staff, was provided an access link to the survey webpage into which they entered the answers to each questionnaire. Participation was on a voluntary basis.

The types of hospitals that participated in the survey include “hospitals”, “general hospitals”, “tertiary hospitals”, and “long-term care hospitals”, the definitions of which are based on the Korean Medical Service Act. Here, “hospital” refers to healthcare facilities with more than 30 beds providing mostly inpatient services. A “general hospital” refers to hospitals with more than 100 beds and at least 7–9 specialized departments. Among these general hospitals, the Minister of Health and Welfare designates “tertiary hospitals” which are training hospitals with at least 20 specialized departments. “Long-term care hospitals” refer to hospitals providing medical services to inpatients in need of long-term care. The survey questionnaire was distributed to a total of 2108 hospitals, which included all “tertiary hospitals” (42), all “general hospitals” (298), all “long-term care hospitals” (1496) in Korea, and “hospitals” operating at least two of the three following functions (272): intensive care unit (ICU), emergency room, and/or operating room. Most of the survey questions were associated with these three functions, so hospitals operating at least two of them were surveyed in order to yield sufficient information.

The survey questionnaire was developed by the government through expert consultations. The expert group consisted of medical doctors and registered nurses with expertise in infection control. The development of the survey questionnaire was largely based on the IPC standards stipulated in the Korean Medical Service Act, which is the law regulating the duties and responsibilities of medical institutions in Korea. For example, the Act stipulates the required number of full-time IPC specialists, the structure of the IPC committee, and the compulsory training of IPC specialists, etc. After rounds of expert discussions, the survey questionnaire was developed to encompass 9 components, which are: (1) IPC teams and programs; (2) IPC committees; (3) IPC costs and expenditures; (4) IPC training and education; (5) IPC surveillance; (6) staffing; (7) equipment and built environment; (8) IPC activities; and (9) IPC activities to prevent antimicrobial resistance. IPCAF, on the other hand, is composed of 8 sections and a total of 81 indicators. Each indicator is associated with scores based on the answers chosen and ultimately added up to yield an aggregate score for each component as well as an overall score. As the KNIPCS questionnaire was not identical to the IPCAF in its structure and scoring system, their direct comparison of attained scores was not possible. However, there were common individual indicators included in both surveys, allowing comparison of the results at an indicator level. The structure of the KNIPCS in comparison to the WHO IPCAF is presented (Table 1).

**Table 1:** The structure of the Korean national IPC survey in comparison to WHO IPCAF

WHO IPCAF (core component)	Questions in the Korean national IPC survey that correlate with indicators in each core component of the WHO IPCAF
Core component 1. IPC program (10 indicators)	Availability of an IPC team Availability of an IPC committee Availability of an IPC program Availability of at least one full-time IPC professional or equivalent^a^ Membership of an IPC team (doctors, nurses, etc.) Structure and operation of the IPC committee
Core component 2. IPC guidelines (8 indicators)	Availability of a guideline for: environmental cleaning, transmission-based precautions, disinfection and sterilization, antibiotic stewardship, etc
Core component 3. IPC education and training (10 indicators)	Availability of personnel to lead IPC training Mode of IPC training^b^ Availability of ongoing education for IPC staff
Core component 4. Healthcare-associated infection surveillance (15 indicators)	Inclusion of HCAI surveillance in the IPC program Availability of personnel responsible for HCAI surveillance Prioritization of HCAI to be targeted for surveillance Surveillance conducted for: Surgical site infections, device-associated infections, MDR pathogens, etc
Core component 5. Multimodal strategies for implementation of IPC interventions (5 indicators)	Inclusion of education and training in IPC programs Discussions of system change (infrastructure, manpower, internal regulations, etc.)^c^
Core component 6. Monitoring/audit of IPC practices and feedback (8 indicators)	Availability of personnel to conduct monitoring/audit Processes monitored: hand hygiene, intravascular catheter insertion, activities to prevent MDR pathogens, disinfection and sterilization
Core component 7. Workload, staffing and bed occupancy (8 indicators)	Staffing levels Bed occupancy (spacing, one patient per bed, etc.)
Core component 8. Built environment, materials, and equipment for IPC at the facility level (17 indicators)	Availability of materials and supplies (personal protection equipment, disposable items, etc.) Availability of isolation rooms Availability of hand hygiene stations

*IPC* infection prevention and control, *WHO* World Health Organization, *IPCAF* Infection Prevention and Control Assessment Framework, *MDR* multi-drug resistant, *HCAI* healthcare-associated infection

^a^A nurse or doctor working full-time in IPC

^b^Using written information, oral instruction, e-learning, interactive training, etc.

^c^However, in the Korean National Infection Prevention and Control survey, these questions were not addressed as a part of a “multimodal strategy” as defined in the WHO IPCAF, but as an independent indicator

The execution of the national survey was in accordance with article 17 of the Infectious Disease Control and Prevention Act, which stipulates the role of the government in conducting surveys regarding the conditions and status of infection control. As the survey did not include any individual human data, ethics approval was waivered. Nevertheless, data was collected and analyzed by a third party, a statistical analysis company contracted prior to the initiation of the survey, in order to ensure privacy and data protection.

## Results

A total of 1442 hospitals completed the survey, with a response rate of 68.4%. The general characteristics of the participating hospitals are provided in Additional file 1. Concerning CC1, all tertiary hospitals and the majority of general hospitals had an IPC team (96.5%) as well as an IPC committee (98.8%) with at least one full-time IPC professional (Table 2). Among hospitals, 112 (67.0%) had at least one full-time IPC professional and 56 (33.5%) had an IPC program. The average number of IPC staff in tertiary hospitals was 4.7 for doctors and 7.3 for nurses. In general hospitals, the IPC team consisted of 1.6 doctors and 2.2 nurses on average. A total of 87 (33.4%) general hospitals held two or fewer committee meetings per year, and 23 (8.8%) answered that committee meetings were not helpful in the actual implementation of IPC programs in the facility. In tertiary hospitals and general hospitals, more than 80% of IPC training (CC3) was provided by oral instruction, whereas only 25.2% of tertiary hospitals and 14.9% of general hospitals used an interactive mode of training. Doctors in the IPC team received more than 20 h of ongoing education, and nurses received more than 40 h on average in both tertiary hospitals and general hospitals.

**Table 2:** Results of the Korean national IPC survey corresponding to CC1 and CC3 of the WHO IPCAF

Korean national IPC survey questions	Answer	Number (%)			
		Tertiary hospital (n = 42)	General hospital (n = 260)	Hospital (n = 167)	Long-term care hospital (n = 973)
*Corresponding IPCAF core component: Core Component 1 (IPC program)*					
Availability of an IPC team	Yes (Available)	42 (100.0)	251 (96.5)	37 (22.1)	62 (6.3)
Availability of an IPC committee	Yes (available)	42 (100.0)	257 (98.8)	64 (38.3)	704 (72.4)
Number of IPC committee meetings held per year	0–1	0 (0.0)	4 (1.5)	6 (9.4)	35 (5.0)
	2	6 (14.3)	83 (31.9)	22 (34.4)	296 (42.0)
	3 times or more	36 (85.7)	170 (65.3)	36 (21.5)	373 (38.3)
Do you feel that committee meetings are helpful?	Yes	42 (100.0)	234 (90.0)	48 (28.7)	630 (64.7)
	No	0 (0.0)	23 (8.8)	16 (9.5)	74 (7.6)
	Not replied	0 (0.0)	3 (1.2)	103 (61.8)	269 (27.7)
Availability of an IPC program	Yes (available)	42 (100.0)	249 (95.8)	56 (33.5)	745 (76.6)
Availability of at least one full-time IPC professional	Full-time IPC nurse	42 (100.0)	255 (98.1)	112 (67.0)	DNA
Average number of doctors and nurses in the IPC team (number)	Doctors	4.74	1.66	0.91	0.68
	Nurses	7.31	2.23	0.95	1.00
*Core Component 3 (IPC education and training)*					
Mode of IPC training (percentage to total number of training sessions, multiple answers allowed)	Oral instruction	88.9	86.7	77.0	61.6
	e-learning	9.8	9.5	13.9	37.2
	Interactive	25.2	14.9	8.1	12.3
Ongoing education for IPC staff (average hours of received education per year)	Doctors	21.67	21.26	5.20	6.57
	Nurses	44.82	46.10	12.97	12.15

*IPC* infection prevention and control, *CC* core component*, WHO* World Health Organization, *IPCAF* Infection Prevention and Control Assessment Framework, *DNA* data not available

Regarding CC4, all 42 tertiary hospitals were participating in the Korean National Healthcare-associated Infections Surveillance (KONIS), whereas 90 general hospitals (34.6%) and the majority of hospitals and long-term care hospitals were not (Table 3). Profound differences were revealed concerning surveillance activities among hospital types, as most of the tertiary hospitals (97.6%) and general hospitals (73.8%) were performing prioritization of HCAI and risk factors to be targeted for surveillance, whereas only 29.9% of hospitals were doing so. All tertiary hospitals and the majority of general hospitals were performing surveillance on surgical site infections (69.6%), bloodstream infections (81.4%), urinary tract infections (85.4%), and pneumonia (81.0%), all of which are a part of the KONIS. With regard to CC6, hand hygiene and disinfection/sterilization were the most actively monitored activities in all hospital types, even in long-term care hospitals (98.8% for hand hygiene, 85.3% for disinfection/sterilization). Compared to these activities, the monitoring and audit of activities related to antimicrobial resistance did not show a similarly high percentage, as only 128 (57.7%) of general hospitals performed multi-drug resistant (MDR) pathogen screening before ICU admission.

**Table 3:** Results of the Korean national IPC survey corresponding to CC4 and CC6 of the WHO IPCAF

Korean national IPC survey questions	Answer		Number (%)			
			Tertiary hospital (n = 42)	General hospital (n = 260)	Hospital (n = 167)	Long-term care hospital (n = 973)
*Corresponding IPCAF core component: Core Component 4 (HCAI surveillance)*						
Participation in the Korean National Healthcare-associated Infections Surveillance (KONIS)	Yes	KONIS ICU survey	42 (100.0)	162 (62.3)	5 (3.0)	12 (1.2)
		KONIS SSI survey	42 (100.0)	155 (59.6)	5 (3.0)	5 (0.5)
	No		None	90 (34.6)	160 (95.8)	957 (98.4)
Prioritization/identification of HCAI and risk factors to be targeted for surveillance	Yes		41 (97.6)	192 (73.8)	50 (29.9)	383 (39.4)
Surveillance conducted for:	SSI		42 (100.0)	176 (69.6)	77 (57.0)	46 (4.9)
	Bloodstream infections		42 (100.0)	206 (81.4)	40 (29.6)	114 (12.1)
	Urinary tract infections		42 (100.0)	216 (85.4)	46 (34.1)	238 (25.2)
	Pneumonia		42 (100.0)	205 (81.0)	51 (37.8)	196 (20.7)
*Core Component 6 (Monitoring/audit of IPC practices and feedback)*						
IPC practices monitored:	Hand hygiene		41 (97.6)	235 (92.9)	121 (89.6)	935 (98.8)
	MDR pathogen screening^a^		35 (83.3)	128 (57.7)	5 (25.0)	4 (25.0)
	Disinfection and sterilization^b^		42 (100.0)	253 (97.3)	108 (64.7)	830 (85.3)
	Isolation of MDR positive patients		42 (100.0)	207 (93.2)	12 (70.6)	6 (54.5)

*IPC* infection prevention and control, *CC* core component,* WHO* World Health Organization, *IPCAF* Infection Prevention and Control Assessment Framework, *HCAI* healthcare-associated infection, *ICU* intensive care unit, *SSI* surgical site infection, *MDR* multi-drug resistant

^a^MDR pathogen surveillance screening before ICU admission

^b^Disinfection and sterilization of medical equipment/instruments

All tertiary hospitals had guidelines (CC2) available for antibiotic stewardship, disinfection and sterilization, and environmental cleaning (Table 4). The majority of general hospitals (≥ 95%) also had these guidelines available, with the exception of antibiotic stewardship (65.7%). Regarding guidelines for disinfection and sterilization, 123 (73.6%) hospitals and 889 (91.3%) long-term care hospitals had them available. Bed spacing (CC7) in ICUs was an average of 1.8 m for tertiary hospitals and 1.6 m for general hospitals. With regards to CC8, the average number of patients sharing a hand hygiene station was lowest in tertiary hospitals (1.6 persons) and highest in long-term care hospitals (6.8 persons). Personal protection equipment, such as masks and alcohol-based hand rubs was widely available in all hospital types. Tertiary hospitals (90.5%) and general hospitals (67.7%) used sterile compounds or separate preparation areas to prepare fluids, whereas 49.5% of long-term care hospitals did so in other areas where sterility was not guaranteed.

**Table 4:** Results of the Korean National IPC survey corresponding to CC2, CC7, and CC8 of the WHO IPCAF

Korean national IPC survey questions	Answer	Number (%)			
		Tertiary hospital (n = 42)	General hospital (n = 260)	Hospital (n = 167)	Long-term care hospital (n = 973)
*Corresponding IPCAF core component: Core Component 2 (IPC guidelines)*					
Availability of a guideline for:	Antibiotic stewardship	42 (100.0)	171 (65.7)	86 (51.5)	302 (31.0)
	Disinfection and sterilization^a^	42 (100.0)	251 (96.5)	123 (73.6)	889 (91.3)
	Environmental cleaning	42 (100.0)	249 (95.8)	120 (71.9)	DNA
*Corresponding IPCAF core component: Core Component 7 (Workload, staffing and bed occupancy)*					
Bed spacing in ICU (meters)	Average distance between patient beds	1.8	1.6	DNA	DNA
*Core Component 8 (Built environment, materials, and equipment for IPC)*					
Hand hygiene stations in ICU	Number of patients sharing a hand hygiene station	1.6	2.8	4.1	6.8
PPE in ICU (percentage among hospitals with ICU)	Masks	42 (100.0)	221 (99.5)	17 (85.0)	16 (100.0)
	Alcohol-based hand rub	42 (100.0)	221 (99.5)	20 (100.0)	16 (100.0)
Isolation rooms in ER	Average number of negative pressure isolation rooms in ER	2.4	0.6	DNA	DNA
Injection safety: area where fluid/injections are prepared^b^	Sterile compound/clean rooms	10 (23.8)	9 (3.5)	1 (0.6)	11 (1.1)
	Dedicated/separate preparation area in wards	28 (66.7)	167 (64.2)	116 (69.5)	483 (49.6)
	Other areas^c^	4 (9.5)	84 (32.3)	50 (29.9)	479 (49.3)

*IPC* infection prevention and control, *CC* core component,* WHO* World Health Organization, *IPCAF* Infection Prevention and Control Assessment Framework, *DNA* data not available (due to the small number of hospitals that operate either ER and/or ICU in this hospital group), ICU intensive care unit, *PPE* personal protection equipment, *ER* emergency room

^a^Disinfection and sterilization of medical equipment/instruments

^b^Indicator exclusive to the Korean National Infection Prevention and Control survey but relevant to the core component 8 of IPCAF

^c^Shared, a non-dedicated area in wards, bedsides, etc.

## Discussion

The WHO IPCAF was based on the WHO CCs [2], which is an evidence-based guideline on the implementation of essential components for IPC programs in terms of effectiveness in reducing HCAI at the facility level. The 8 essential CCs address the complex nature of IPC, encompassing technical guidelines, human resources, surveillance, and the built environment. Therefore, analysis of the national survey in comparison to the WHO IPCAF allowed a comprehensive exploration of the status and gaps in IPC programs in Korean hospitals.

Much evidence shows that an IPC structure, composed of a dedicated IPC team and relevant in-house governance, reduces HCAIs and is thus the single most important component in an institution’s IPC capacity [7, 8]. In general, the results of this study show that these essential IPC structures are well in place in most tertiary and general hospitals in Korea. This may be partly due to relevant legislative measures, such as the Korean Medical Service Act, which mandated the requirement for an IPC committee and an IPC team in all general hospitals and hospitals with more than 150 beds. Studies show that countries with similar legislative regulations have a more robust implementation of IPC structures compared to countries that do not, suggesting that the influence of relevant legislation is critical in the establishment of IPC components in healthcare facilities [9, 10]. However, it is worth noting that in Korea, such legislative measures were coupled with various policy measures to promote the quality of care in IPC. For example, implementation of IPC programs was included or expanded in performance evaluation programs such as the Korean Healthcare Accreditation System and healthcare quality evaluation. Also, a novel reimbursement scheme was developed within the National Health Insurance in 2016 that pays hospitals a certain amount of fee per patient’s admission day provided that the hospital meets specified criteria, which include the operation of an IPC team with a designated number of full-time, trained IPC staff and the development of an IPC program, etc. It is assumed that a combined effect of legislation and such quality-driven approaches has resulted in a generally high percentage of IPC teams and programs in tertiary and general hospitals in Korea.

On the other hand, the percentage of IPC team availability was relatively low among long-term care hospitals (6.3%) and hospitals (22.1%). These hospital groups were not subject to the mandatory implementation of IPC teams and committees at the point of the survey, which may partly explain the low percentage of IPC teams and other CC1 components. Long-term care hospitals in Korea, which are mostly privately owned and offer health services related to chronic diseases and other geriatric illnesses, are not required by law to have IPC teams. This legal exemption is primarily due to the operational difficulties of long-term care hospitals, mainly related to insufficient budget and manpower [11]. However, given that recent research indicates that long-term hospitals are vulnerable to HCAI [12], it is clear that stronger policy support is required to empower long-term hospitals in infection control.

It is interesting to note that despite the high percentage of hospitals with an established IPC team and committee, their actual operations were quite heterogeneous and suboptimal in many hospitals, implying that the installation of these IPC structures does not instantly guarantee effective execution of IPC activities. One example is shown through the data on the IPC committee meeting. IPC committee meetings, albeit being the main decision-making process concerning IPC policies in hospitals, are not actively carried out or not considered helpful in 61.8% of the hospitals. Similar results were revealed in another study, in which 23% of hospitals answered that the IPC committee was not supported by senior staff [10]. It is considered that further technical support, such as training and education targeting hospital executives, is warranted for such essential IPC structures to effectively function.

IPC education and training has proven to be effective in reducing HCAI if conducted effectively to achieve behavior change [13–15]. In addition, as IPC is relevant to all healthcare workers, IPC education has to target not only the IPC specialists and frontline workers but also all general staff in the facility. Results of this study reveal that IPC education targeting IPC specialists was one of the components with generally high compliance, possibly owing to the Korean Medical Service Act that stipulates mandatory education of at least 16 h for members of the IPC team. The Act mandates that official IPC education to IPC team members should be provided by the government, government-funded institutions (e.g., Korea Human Resource Development Institute for Health and Welfare), professional associations, and/or academic societies. The Act also specifies specific training topics and subjects to guarantee the quality of education provided.

On the other hand, IPC education targeting the general staff in the facility is currently roughly regulated as one of the responsibilities of the hospital manager and/or IPC teams in the hospital. Therefore, convenient, one-way oral instruction was more frequently applied compared to interactive training in all hospital groups. Previous studies conducted among hospitals in Austria and Germany also showed that interactive training was the least utilized mode of training [9, 10]. Evidence suggests that participatory, interactive IPC education involving task-based strategies and simulation is associated with decreased HCAI and is therefore strongly recommended through the WHO IPC CC guideline [2, 13–15]. Future policies should aim to support IPC education targeting all staff in the facility and also strengthen various modes of effective training, such as interactive learning.

HCAI surveillance (CC4) of Korea centers around KONIS, which is the government-led surveillance program in which hospitals participate on a voluntary basis.KONIS data reveals that major HCAI rates in Korea have been decreasing over the past decade. Urinary tract infections have decreased from 4.24 cases per 1000 patient days in 2006 to 0.88 cases per 1000 patient days in 2016. Pneumonia in ICUs has decreased from 3.68 cases per 1000 patient days in 2006 to 1.00 cases per 1000 patient days in 2016 [16]. KONIS mainly surveys IPC rates in ICUs and surgical site infection rates, which explains the relatively high participation in the tertiary and general hospital groups that operate ICUs and operating rooms.

KONIS applies standardized data collection methods (IPCAF CC4 question 9), informatics (IPCAF CC4 question 4), protocols (IPCAF CC4 question 8), audit processes (IPCAF CC4 question 10), uniform data feed-back methods (IPCAF CC4 question 14), and governance (IPCAF CC4 question 3), which all participating hospitals share. In contrast to the high participation in tertiary and general hospitals, only a limited number of hospitals in the hospital and long-term care hospital groups participated in the KONIS, revealing the need to expand IPC surveillance in these hospital groups. The government is in the process of developing surveillance modules for these smaller-sized hospitals and long-term care hospitals [17].

While CC2 was a component with high mean scores in studies from other countries, this study revealed different results. The guideline for disinfection and sterilization was the most commonly available IPC guideline across all hospital types, which may be contributing to the relatively high percentage of hospitals monitoring disinfection and sterilization. On the other hand, the percentage of hospitals with antibiotic stewardship guidelines was quite low, especially in the hospital (51.5%) and long-term care hospital group (31.0%). This result, along with the relatively low percentage of hospitals performing MDR pathogen screening and isolation of MDR-positive patients (CC6), reveal that antimicrobial resistance activities in these hospital groups are suboptimal. Many previous studies have also raised issues regarding the relatively high prevalence of MDR pathogens in smaller-sized hospitals [18, 19]. As the development of the WHO CCs was in response to the global threats posed by antimicrobial resistance, this figure is quite alarming, which calls for more active policy actions regarding AMR [2, 20].

Many of the indicators in WHO CC7 and CC8, such as staffing levels, bed occupancy, power and water supplies, ventilation, waste management, sterile supply department, etc., were developed based on the standards required for healthcare in medium- and low-resource settings [2]. The Korean Medical Service Act stipulates a wide range of basic standards on staffing and the built environment, through which the hospitals’ license to operate is strictly regulated. Therefore, it was appropriate to assume that hospitals in Korea met the minimum standards that the WHO core components suggested. Nevertheless, some indicators that were considered relevant in the local context were included in the national survey and are worth further discussion.

Hand hygiene stations (IPCAF CC8 question 3) were generally accessible to all patients, but the number of patients per station was especially high in long-term care hospitals (6.8) compared to tertiary and general hospitals. The new legal standard requiring at least one hand hygiene station per 3 patients in ICUs went into force in 2017. This standard applies to only newly established facilities, which may explain why the number of patients per station is higher than 3 in the hospital and long-term care hospital group. Such insufficiency in the built environment has been an issue in other high-income countries as well [21], underscoring the need for continued policy support in establishing the basic infrastructure in healthcare facilities. In contrast to this, personal protection equipment (IPCAF CC8 question 10), such as masks and hand rubs, were widely available in all hospital groups. Fluid and injections were prepared in areas other than clean rooms and dedicated preparation areas in 32.3% of general hospitals and 49.3% of long-term care hospitals. As fluids and drugs should be prepared under the strictest of conditions to prevent contamination and possible infection hazards that stem from it, more strict measures are being taken around the world regarding the preparation of pharmaceuticals in hospitals. This implies that national-level policy measures should be further developed and implemented to promote safe preparation and injection practices [22].

Implementation of multimodal strategies (CC5) was not effectively surveyed through the national survey. However, reflecting that CC5 was the component with the lowest mean score in other high-income countries [7, 8], it could be speculated that Korean hospitals may also have similar difficulties in the effective implementation of multimodal strategies. Whereas individual elements of multimodal strategies, e.g. education and training, could already be at work, systematically integrating them to achieve behavioral change requires a certain level of expertise in the field. Policy measures should be put forth to strengthen facilities in delivering multimodal strategies. It is also advisable that future national surveys incorporate indicators relevant to multimodal strategies to allow exploration of their status in Korean hospitals.

This study has several limitations. First of all, the national survey was conducted on a voluntary basis, and therefore, facilities with a high interest in IPC may be overrepresented. Another limitation is that although the survey was roughly pre-tested in two randomly selected hospitals, it did not go through an official pilot test due to time constraints and logistical reasons. Also, as the answers were self-administered, the data can be different from other currently available national-level data sets, such as the national health insurance data. Lastly, although many of the survey questions were similar to their IPCAF counterparts, the answering options were different and may have hampered direct comparison.

## Conclusions

The first-ever nationwide survey of IPC and the evaluation of its results in comparison to the WHO IPCAF revealed an overview of IPC status in Korean hospitals and provided valuable policy insights. The establishment of a basic IPC structure, such as full-time IPC staff and IPC programs, is generally high in tertiary hospitals and general hospitals, which is speculated to be the effect of both legislative and quality-based policy measures in IPC. The establishment of the IPC structure was relatively weak in long-term care hospitals and smaller-sized hospitals, which calls for future policy efforts targeting these hospital groups. Although hospitals were actively conducting the individual elements of multimodal strategies, whether they were implemented in a coordinated and systematic manner should be more actively explored in future surveys.

## Supplementary Information

**Additional file 1**. Table e1. General characteristics of 1,442 hospitals in the Korean national IPC survey.

## Data Availability

All relevant data and materials are included in the main text and the supplementary information file of this manuscript.

## Abbreviations

IPC: Infection prevention and control
IPCAF: Infection Prevention and Control Assessment Framework
KNIPCS: The Korean National Infection Prevention and Control Survey
HCAI: Healthcare-associated infection
WHO: World Health Organization
CC: Core components
ICU: Intensive care unit
KONIS: Korean National Healthcare-associated Infections Surveillance
MDR: Multi-drug resistant
