# Supplementary material for: A nationwide survey on the implementation of infection prevention and control components in 1442 hospitals in the Republic of Korea: comparison to the WHO Infection Prevention and Control Assessment Framework (IPCAF)
Source: Antimicrob Resist Infect Control. 2022 May 13;11:71. doi: 10.1186/s13756-022-01107-w (PMC9101985; doi:10.1186/s13756-022-01107-w)
Supplement: Supplementary file 1 — Additional file 1. Table e1. General characteristics of 1,442 hospitals in the Korean national IPC survey. [file 13756_2022_1107_MOESM1_ESM.docx]

**Additional file 1**

Table e1. General characteristics of 1,442 hospitals in the Korean national IPC survey.

| Characteristics |  | Tertiary hospital  (n=42) | General hospital  (n=260) | Hospital  (n=167) | Long-term care hospital  (n=973) |
| --- | --- | --- | --- | --- | --- |
| Response rate (%) |  | 100.0 | 87.2 | 61.4 | 65.0 |
| Location | Seoul and greater Seoul metropolitan area | 21(50.0) | 105(40.3) | 50(29.9) | 295(30.3) |
|  | Other | 21(50.0) | 155(59.7) | 117(70.1) | 678(69.7) |
| Number of hospital beds | Less than100 | - | - | 59(35.3) | 104(10.7) |
|  | 100~200 | - | 27(10.3) | 67(25.7) | 612(62.9) |
|  | 201~400 | - | 154(59.2) | 36(13.8) | 213(21.9) |
|  | More than 401 | 42(100.0) | 79(30.3) | 5(1.9) | 44(4.5) |
